# Effect of Nano- and Microzinc Supplementation on the Mineral Composition of Bones of Rats with Induced Mammary Gland Cancer

**DOI:** 10.3390/foods12061348

**Published:** 2023-03-22

**Authors:** Dorota Skrajnowska, Jakub Idkowiak, Arkadiusz Szterk, Karol Ofiara, Kinga Augustyniak, Barbara Bobrowska-Korczak

**Affiliations:** 1 Department of Toxicology and Food Science, Faculty of Pharmacy, Medical University of Warsaw, Banacha 1, 02-097 Warsaw, Poland; dorota.skrajnowska@wum.edu.pl (D.S.); kingaaugustyniak3@gmail.com (K.A.); 2 Department of Analytical Chemistry, Faculty of Chemical Technology, University of Pardubice, Studentská 573, CZ-532 10 Pardubice, Czech Republic; jakubidkowiak1@gmail.com; 3 Transfer of Science sp. z o. o., Strzygłowska 15, 04-872 Warsaw, Poland; a.szterk@transferofscience.com (A.S.); k.ofiara@transferofscience.com (K.O.)

**Keywords:** nanozinc, microzinc, bone, mineral metabolism, breast cancer

## Abstract

Background: The aim of this study was to determine changes in the mineral composition of the bones of rats with chemically induced mammary gland cancer and to attempt to establish whether a specific diet modification involving the inclusion of zinc ions in two forms—nano and micro—will affect the mineral composition of the bones. Methods: Female Sprague–Dawley rats were used for the research. The animals were randomly assigned to three experimental groups. All animals were fed a standard diet (Labofeed H), and selected groups additionally received zinc nanoparticles or microparticles in the amount of 4.6 mg/mL. To induce mammary cancer, the animals were given 7,12-dimethyl-1,2-benz[a]anthracene. The content of Ag, As, B, Ba, Cd, Cr, Cu, Mn, Ni, Pb, Rb, Se, Sr, Tl, U, and V was determined using ICP-MS, while that of Ca, Fe, K, Mg, Na, and Zn was determined using FAAS. Results: The use of a diet enriched with zinc nano- or microparticles significantly influenced the content of the elements tested. In the bones of rats fed a diet with zinc nanoparticles, changes were found in the content of Ca, Mg, Zn, Cd, U, V, and Tl, while in the case of the diet supplemented with zinc microparticles, there were differences in six elements—Ca, Mg, B, Cd, Ag, and Pb—compared to animals receiving an unsupplemented diet. Conclusions: The content of elements in the bone tissue of rats in the experimental model indicates disturbances of mineral metabolism in the tissue at an early stage of mammary cancer.

## 1. Introduction

Cancer and the treatments applied can have profound effects on bone health [1,2,3]. During the course of cancer, a loss of bone mass takes place, leading to fractures, hypercalcaemia, pain, or a decline in mobility [1,2]. The loss of bone mass in cancer patients is multifactorial, resulting from the disease itself, treatment, and age [3,4]. For this reason, care for bone health is an important and increasingly valued element of comprehensive care for cancer patients. The composition of micro- and macroelements is often disturbed in the conditions of the neoplastic process, but there are almost no studies on the relationship between the levels of elements in bone tissue at the initial and later stages of the disease [5,6,7,8,9,10].

A mineral whose role should be particularly emphasized is zinc in its classic macro form, as an anticancer factor that also plays a major role in bone metabolism [9,10,11,12,13,14,15,16]. Zinc is an essential mineral for normal skeletal growth and bone homeostasis. Studies with humans indicate a link between reduced zinc concentrations and impaired bone parameters such as mineral density and the synthesis of bone turnover markers [13,17,18]. Zinc, as a cofactor of alkaline phosphatase, is involved in the mineralisation of the bone extracellular matrix, promotes bone regeneration, stimulates osteoblast proliferation, participates in osteoclastogenesis, chondrogenesis, and the activation of aminoacyl-tRNA synthetase, and increases the production of IGF-1 and TGF-b1—growth factors in bone cells [13,14,19,20,21]. As zinc is well absorbed and likely tolerated, it is possible to increase zinc concentration in areas requiring bone regeneration by even taking zinc supplements orally [13].

It is unknown how and to what degree a micro or nano form of zinc will modify bone composition in the conditions of the neoplastic process. The nano form in particular alters the bioavailability of zinc, from absorption to distribution and finally to its utilization in cells, which may translate into different effects in bone tissue, which is relatively stable. In comparison with macro- or microscale materials, nanoparticles exhibit differences in size, specific surface area, chemical and mechanical reactivity, optical, electrical, and magnetic properties, and even the appearance of quantum effects [22]. The specific heterogeneity of the surface and its area are also important in terms of other characteristics, including activity [23]. Research on zinc in nano form is now particularly important because zinc increasingly appears in this form in food, plastics, personal hygiene products, and disease therapies, e.g., cancer treatment [22,23,24,25].

There are numerous reports concerning zinc oxide nanoparticles as effective anti-tumour agents [24,26]. Their main advantage is their preferential cytotoxicity against cancer cells in vitro: 28–35 times the selective toxicity towards cancer cells compared with that of normal cells [27,28]. Cancer cells often contain high concentrations of phospholipid anions in the cell membrane, thus showing high membrane potentials. It is suggested that positively charged ZnO nanoparticles induce selective cytotoxicity against cells located in negatively charged sites in the tumour [29,30]. Another important advantage of ZnO nanoparticles is that their size can be easily modified. Most in vitro studies show the maximum cellular uptake of nanoparticles in a size range of 10–60 nm, irrespective of the composition of the core or the surface charge [31]. Another very important characteristic for cancer treatment is the ability to produce reactive oxygen species (ROS), which lead to the death of the tumour cell [29]. Increased ROS production additionally causes mitochondrial dysfunction and activation of apoptosis and necrosis, as well as an increase in oxidative DNA damage and mitotic catastrophe. Wahab et al. [32] observed that ZnO NPs at a very small dose of 25 µg/mL induced cytotoxicity and apoptosis in MCF-7 and HepG2 cancer cells (mammary and liver cancer, respectively). Apoptotic cell death resulting from the induction of oxidative stress was characterized by upregulation of the protein p53 and caspase 3, and downregulation of the anti-apoptotic gene Bcl2.

It is not known whether the distribution of elements from and to bone tissue, which is an enormous deposit of minerals for the growing tumour, changes significantly in the conditions of the neoplastic process, or to what degree these change are modified by diet [33].

The aim of this study was to determine differences in the mineral composition of the bones of rats with chemically induced mammary cancer and to establish whether a specific diet modification involving the inclusion of zinc ions in two forms—nano and micro—would affect the mineral composition of the bones. The weight of the bones was determined as well as the content of 22 elements, silver (Ag), arsenic (As), barium (Ba), boron (B), calcium (Ca), cadmium (Cd), chromium (Cr), copper (Cu), iron (Fe), potassium (K), magnesium (Mg), molybdenum (Mn), sodium (Na), nickel (Ni), lead (Pb), rubidium (Rb), selenium (Se), strontium (Sr), thallium (Tl), uranium (U), vanadium (V), and zinc (Zn), in the femurs of rats with induced mammary gland tumours, fed three different diets: without supplementation, supplemented with zinc microparticles, and supplemented with zinc nanoparticles.

## 2. Materials and Methods

### 2.1. Laboratory Animals

Approval for the experiment was obtained from the Animal Ethics Committee of the Faculty of Biology, University of Warsaw (approval no. 645/2018 issued on 3 July 2018). Twenty-four female Sprague Dawley rats at the age of 4 weeks were obtained from the Laboratory of Experimental Animals of the Department of General and Experimental Pathology, Medical University of Warsaw. All rats were fed a standard diet (Labofeed H, Kcynia, Poland) and had ad libitum access to tap water. The rodents were kept in a room with a constant temperature of 22 °C with a 12 h light/dark cycle. After a 10-day acclimation period, the animals were randomly assigned to three groups: a control group on a standard diet (s), supplementation with zinc in microscale (m), and supplementation with zinc in nanoscale (n). Each group contained 8 rats. Rats from the m group received 0.4 mL of an aqueous suspension of zinc microparticles (342 nm) in a dose of 4.6 mg/mL via gastric tube. In the same manner, rats in the n group received 0.4 mL of an aqueous suspension of zinc nanoparticles (99 nm) in an amount of 4.6 mg/mL. Interactive factors were eliminated by applying the same experimental procedure to all rats, i.e., age, experimental time, feed, housing conditions, tumour induction method, and supplementation method. The control group (s) was given 0.4 mL of water instead of zinc to induce a similar level of stress to the animals in the control group. Zinc compounds were administered to the animals from the age of 40 days to 20 weeks. The zinc level in the diet was twice that of the level in the standard diet, i.e., 76.9 mg/kg of diet. The experiment lasted 100 days. The cumulative zinc intake is presented in Table 1. The procedure for synthesizing the micro and nano forms of zinc is presented in our previous article [34].

The Labofeed H diet contained following other compounds (per 1 kg): protein (210 g), fat (39.2 g), fibre (43.2 g), ash (55 g), carbohydrates (300 g), vitamin A (15,000 IU), vitamin D3 (1000 IU), vitamin E (90 mg), vitamin K3 (3 mg), vitamin B1 (21 mg), vitamin B2 (16 mg), vitamin B6 (17 mg), vitamin B12 (80 μg), pantothenic acid (30 mg), folic acid (5 mg), nicotinic acid (133 mg), P (8.17 g), Mg (3 g), K (9.4 g), Na (2.2 g), Cl (2.5 g), S (1.9 g), Mn (100 mg), Cu (21.3 mg), Co (2.0 mg), I (1.0 mg), and Se (0.5 mg).

### 2.2. Cancer Induction

Mammary cancer was chemically induced in all animals by administering two doses of DMBA (7,12-dimethyl-1,2-benz[a]anthracene; Sigma-Aldrich, St. Louis, MO, USA) dissolved in rapeseed oil. The first dose, 80 mg/kg BW, was administered at 60 days of age, and the second, 40 mg/kg BW, at 90 days of age.

### 2.3. Determination of Levels of Elements

All solvents and reagents were of the highest commercially available purity. Ultrapure water (resistance 18 MΩ cm^−1^), used to prepare all standards and solutions of samples, was obtained from the Barnstead NANOpure Diamond UV system. Samples were dissolved using 65% HNO3 and 37% HCl, Suprapur (Merck, Darmstadt, Germany). Multi-element solutions of Ag, As, B, Ba, Ca, Cd, Cr, Cu, Fe, Mg, Mn, Ni, Pb, Rb, Se, Sr, Tl, U, V, and Zn, each at a concentration of 10 mg/L, were purchased from Inorganic Ventures (Christiansburg, USA). Standard stock solutions of Ca, Fe, K, Mg, Na, and Zn at a concentration of 1000 mg/L were purchased from Merck (Germany). The purity of the plasma gas (argon) and collision cell gas (helium) was above 99.999%.

### 2.4. Sampling

The material for analysis was the femurs of the rats. Following resection, the bones were cleaned of soft tissues, i.e., the joint capsule and muscles, and then frozen at −80 °C. Directly before analysis, the samples were thawed and dried for 10 h at 120 °C, then subjected to mineralization. Each sample of rat bone was placed directly in a hermetically sealed vessel, and 1 mL of HCL and 4 mL of HNO3 were added. Samples were digested in a high-pressure laboratory microwave (Milestone UltraWAVE T640). The heating program was carried out in two steps. In the first step, the temperature was increased linearly from 25 °C to 210 °C over 15 min, and in the second step, the temperature was maintained at 210 °C for 8 min. Following digestion, the samples were diluted with water to a final volume of 100 mL.

### 2.5. Instrumental Analysis

A 7800 quadrupole ICP-MS (Agilent Technologies, Minato City, Tokyo, Japan), equipped with an octopole collision cell, was used for all trace elements analysed. Measurements were made using nickel sampler cones and a skimmer.

Measurements of Ca, Fe, K, Mg, Na, and Zn—at high concentrations of elements—were made using flame atomic absorption spectrometers—Solar GF Zeeman and iCE3500 (Thermo Fisher Scientific 168 Third Avenue Waltham, MA USA) —equipped with lamps with a single hollow cathode, using an air/acetylene flame for the determination of Fe, K, Mg, Na, and Zn and a nitrous oxide/acetylene flame for Ca. The wavelengths for monitoring Ca, Fe, K, Mg, Na, and Zn were 422.7, 248.3, 766.5, 285.2, 589.0, and 213.9 nm, respectively.

Before multielement analysis using ICP-MS and atomic absorption spectrometers, the analytical methods were checked using certificate material (water matrix reference material: EnviroMAT waste water, high (EU-H), catalogue number 140-025-138, lot number S160225019 from SCP Scienc).

### 2.6. Statistical and Bioinformatic Analysis

All calculations were performed in R (v 4.1.2), a free software environment for statistical computing and graphics, or with the use of the MetaboAnalyst platform (v 5.0) (https://www.nature.com/articles/s41596-022-00710-w (accessed on 1 June 2021)). Figures were additionally processed in the Inkscape professional quality vector graphics software (v 1.1). Medians, interquartile ranges (IQR), mean values, and standard deviations (SD) within each group were calculated using the rstatix library (v 0.7.0). For the comparison of all groups, first, the Kruskal–Wallis test was performed. If the Kruskal–Wallis test indicated a statistically significant difference between groups, Dunn’s post hoc test was performed to determine which groups differed (rstatix library). At each step, all obtained *p*-values were corrected using the Benjamini–Hochberg FDR approach (rstatix library). Adjusted box plots for skewed distributions were generated via the ggplot2 library (v 3.3.5) and the litteR library (v 0.9.1) [35]. The principal component analysis was performed using the Metaboanalyst platform (accessed on 24 July 2022). The data were log-transformed and Pareto-scaled before the PCA analysis.

## 3. Results

The results of body and femur weights of rats from the group receiving only a standard diet (without supplementation) and the groups receiving diets supplemented with zinc micro- or nanoparticles are presented in Table 2.

The weights of the femurs of rats receiving diets supplemented with zinc micro- or nanoparticles were higher those in group receiving only a standard diet (without supplementation) (*p* = 0.05)

**Figure 1:**
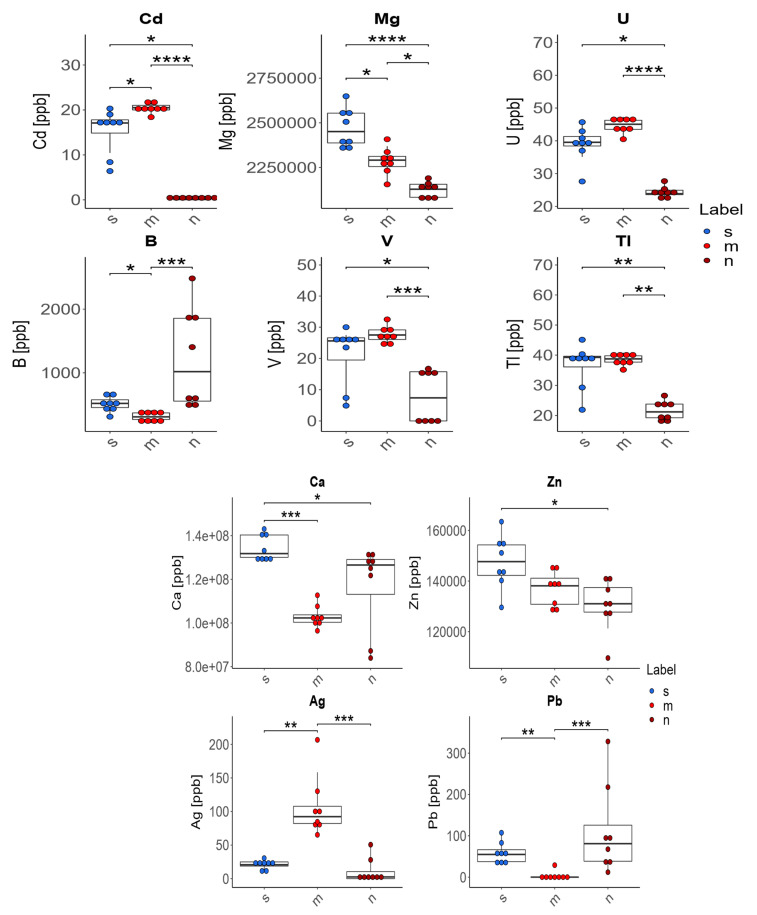
Analysis of concentrations of elements in three experimental groups: s—standard group (blue boxplot); m—group receiving zinc in microparticles (red boxplot); n—group receiving zinc in nanoparticles (dark red boxplot); *p*-value: <0.0001 ****, 0.0001–0.001 ***, 0.001–0.01 **, 0.01–0.05 *.

The content of 22 elements, Ag, As, Ba, B, Ca, Cd, Cr, Cu, Fe, K, Mg, Mn, Na, Ni, Pb, Rb, Se, Sr, Tl, U, V and Zn, in the femurs of rats with induced mammary gland tumours, without supplementation, supplemented with zinc microparticles, and supplemented with zinc nanoparticles was determined, and is presented in Figure 1 and Table 3. Statistically significant changes were noted in the case of 10 elements: Ag, B, Ca, Cd, Mg, Pb, Tl, U, Zn, and V. The following statistically significant relationships were obtained (Figure 1):

*Comparison of the rats from the group receiving zinc microparticles (m) with the group receiving only a standard diet (without supplementation) (s) and the group receiving a diet supplemented with zinc nanoparticles (n):*

− In the case of four of the elements tested (Ag, Cd, Pb, B), supplementation of the diet of rats with zinc microparticles (m) caused significant changes in their concentration in comparison to both the control group on a standard diet (s) and the group receiving zinc nanoparticles (n). The content of Ag and Cd in the bones of these rats was higher and the content of Pb and B was lower than in rats receiving a diet with no supplement or with zinc nanoparticles.

*Comparison of the rats from the group receiving zinc microparticles (m) with the group receiving only a standard diet (without supplementation) (s):*

− The supplementation of the diet of rats with zinc microparticles (m) caused a significant decrease in the concentrations of Mg, B, and Ca and an increase for Cd and Ag only in comparison with the standard diet (s).

*Comparison of the rats from the group receiving zinc nanoparticles (n) with the group receiving only a standard diet (without supplementation) (s):*

− The supplementation of the diet of rats with zinc nanoparticles (n) caused significant changes in the distribution of elements in comparison to the standard diet (s)—a decrease for Ca, Mg, Zn, U, V, Cd, and Tl.

*Comparison of the rats from the group receiving zinc microparticles (m) with the group receiving diets supplemented with zinc nanoparticles (n):*

− The supplementation of the diet of rats with zinc microparticles (m) caused a significant increase for V, Tl, and U only in comparison with the group receiving zinc nanoparticles (n).

There were no statistically significant differences in the levels of As, Ba, Cr, Cu, Fe, K, Mn, Na, Ni, Rb, Se, or Sr in the bones of rats depending on the supplementation used. Median and mean values with standard deviations for the content of 12 elements in the bones of rats are presented in Table 3.

**Figure 2:**
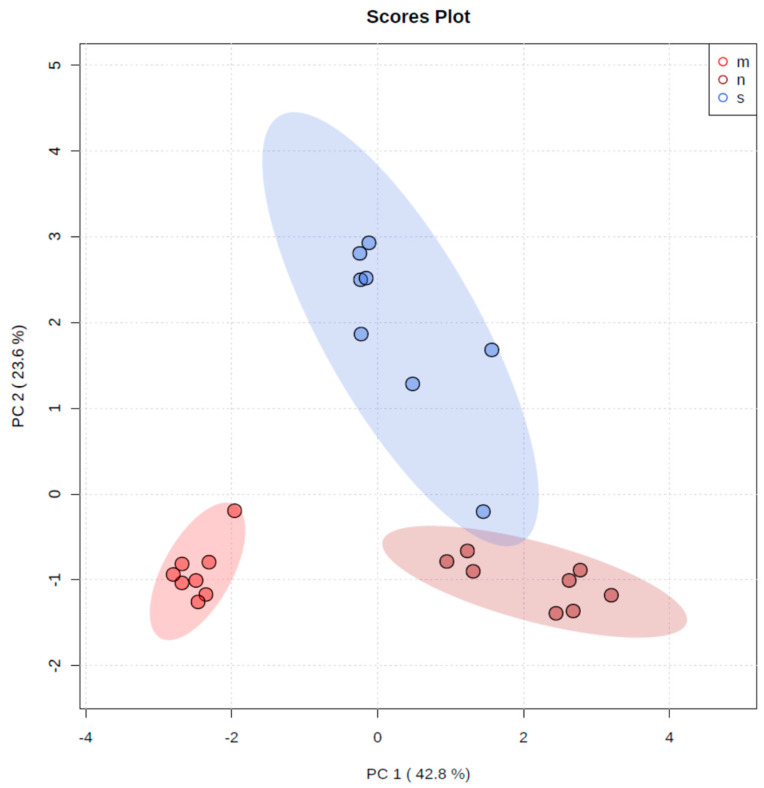
The principal component analysis performed on three groups: s-control, standard diet (blue boxplot); n-nanozinc supplementation diet (dark red boxplot); m-microzinc supplementation diet (red boxplot).

Groups were separated using PCA, especially the group whose diet was supplemented with zinc microparticles from the other two groups, in terms of the composition of elements in the bones in response to the diet modification in conditions of cancer (Figure 2).

## 4. Discussion

Scientific reports on the supplementation of animal feed with metal nanoparticles indicate positive aspects but also stress potential risks [36]. Perceivable advantageous effects include an increase in body weight, increased daily weight gain, and better antibacterial protection. On the other hand, pathological changes may appear in the pancreas, kidneys, liver, small intestine, adrenal glands, or brain [37,38,39,40]. At the cellular level, nanoparticles have been found to induce toxicity, severe inflammation, and cell death. Oral administration of nanoparticles increased the risk of nervous system dysfunction and impairment of cognitive processes in animals. Bąkowski et al. [36] suggest caution in their use in animal production and emphasize the need for further research. Nevertheless, it should be noted that zinc is the second most abundant trace element in the body of animals. It cannot be stored in the body and requires regular intake to meet physiological needs [41]. For this reason, it is often added to food or animal feed in order to meet their daily requirements. Zinc in the form of nanoparticles, due to their better bioavailability and, thus, the possibility of using lower doses, improving growth and antibacterial functions, and modulating immunity and reproduction in animals, seems to be an interesting alternative to conventional sources of zinc. However, there is a need to optimize the dosage and duration of supplementation for both humans and animals, as the results of toxicological studies are not conclusive [22,42].

In the present study, we used supplementation of zinc in reduced forms at both the micro level and in nanoscale, in conditions of chemically induced mammary cancer (DMBA). 7,12-Dimethylbenz(a)anthracene (DMBA) shows selective pro-cancer potential—it induces the formation of mammary gland cancer in particular—which is why it is used as a model chemical carcinogen in research on this type of cancer [43,44,45,46,47]. The tissue analysed in our study was bone tissue, for which zinc is a very important element. The nano form changes the classical bioavailability of zinc, which can translate into the mineral balance of the bone. Mammary cancer can form metastatic lesions in the bones, leading to their destruction. Although dissemination to the bone tissue takes place in most women with advanced breast cancer [48], in the present study, metastasis was not observed in the femurs of rats. DMBA induces the formation of adenocarcinoma of several different morphological types—adenoid cystic carcinoma, in which the tumour cells are separated by small follicles, and papillary carcinoma have been described most frequently. The myoepithelial type of tumour was less common [49]. Interestingly, despite the fact that DMBA-induced tumours were malignant, no metastases were detected in the study by Barros et al. [48]. The authors believe that perhaps a protective factor against this phenomenon is the fact that both epithelial and myoepithelial cells divide extensively, whereas in human breast cancer, proliferation involves almost exclusively epithelial cells [49]. However, there are reports that the appearance of breast tumours can negatively affect the structure of bones (lower mineral density and mechanical strength and poorer structural parameters) even if there is no metastasis to this tissue [33]. It is likely that prior to ‘colonizing’ the bones, breast cancer cells release factors which stimulate resorption of bone tissue, thereby acquiring factors necessary for their development, and at the same time, increase the susceptibility of this tissue to later invasion by the tumour. In our previous study [50], we described the carcinogenic effects of the administration of DMBA and supplementation with various forms of zinc. We found that the supplementation of the diet of rats with zinc nanoparticles ultimately inhibits the formation of cancer tumours (histopathologically—grade 1 tumours and inflammatory infiltration with numerous lymphocytes around the tumour). Despite the fact that the tumours appeared the fastest in that group (in week 16 in all rats), their growth was inhibited over time and with continued supplementation (low final tumour weight of 0.01–1.79 g), and partial remission occurred (incidence 88%, number of tumours per rat 0–3 (1.75 ± 1.04)). In rats receiving a standard diet, the incidence of cancer was 100% and the final weight of the tumours (at 20 weeks) was significantly higher, from 0.1 to 7.8 g. Similar results were obtained in rats whose diet was supplemented with zinc microparticles (incidence 100%, tumour weight from 0.06 to 7.41 g). Grade 2 adenocarcinoma was observed in both the control group on the standard diet and the group receiving zinc microparticles.

In the present study, we supplemented the diet of rats with zinc in the form of nano- and microparticles with double the optimum amount in a standard diet—Labofeed H. Despite this, there was a slight decrease in zinc content in the bones of rats receiving zinc nanoparticles, with no changes noted in the case of supplementation with zinc microparticles in comparison to the standard diet. In the case of other elements, the changes in the levels of Mg and Ca are interesting—irrespective of the form of supplementation, the levels of these elements declined in the bones in comparison with the group that did not receive a supplement (standard control diet). This phenomenon seems very concerning, as these are macroelements that play an important role in the mineralization of the skeleton. This may indicate the intensification of bone mineralization disorders due to ‘preparation’ for metastasis as well as to the supplementation, especially given that the most common symptom of tumour-induced bone lysis is a loss of calcium from the bone.

Calcium plays a key role in bone structure. As much as 99% of the calcium in the body is present in the form of hydroxyapatite, the main mineral component of bones and teeth. A calcium deficiency in the diet leads to a reduction in the mineral content and density in bones, and a long-term deficiency can lead to rickets, osteomalacea, and osteoporosis [51]. Although the primary role of calcium in bone metabolism is the formation of hydroxyapatite, it also performs numerous complex regulatory functions [52]. The literature contains a large amount of data on hypercalcaemia, which is the most commonly diagnosed electrolyte disorder in patients with malignant tumours. It may occur in up to 30% of patients and is usually associated with osteolysis and a poor prognosis [53,54]. The phenomenon of metastasis from the site of the primary tumour through the blood vessels to the bones leads to destruction of the bone tissue and, thus, to the release of large amounts of stored calcium [1,55,56]. This process most likely involves factors activating osteoclasts, such as interleukin 1α, lymphotoxin, TGF-α, TNF-ß, and interleukin-6, leading to excessive bone tissue resorption and, thus, to an increase in the calcium level in the blood [57,58,59,60]. Tumour cells in breast cancer patients produce factors that stimulate osteoclast formation. Osteoclastic bone resorption leads to the release of growth factors from the bone matrix, and these stimulate the further development of the tumour in what can be called a ‘vicious circle’. In the present study, changes in calcium content in the bone tissue seem to be independent of the form of zinc used, although they were more pronounced during supplementation with zinc microparticles. Unfortunately, the results cannot be compared to the findings of other authors. The available literature contains no studies on changes in the bones induced by diet supplementation with nano- or microparticles of zinc in conditions of tumour induction. It is likely that it is the neoplastic process in the mammary gland that causes the changes in bone tissue, despite the absence of metastasis to this tissue (as indicated by the absence of visible morphological characteristics or a decline in femur weight, normal mobility of the rats, and the absence of pain symptoms). If we assume that the changes observed may be the first sign of disturbances in bone mineralization due to ‘preparation’ for metastasis, the addition of zinc in nano or micro form seems to stimulate these unfavourable phenomena. Zachick et al. [61] also showed reduced content of Ca and Mg in bone tissue during Ewing’s sarcoma—a primary bone tumour.

The content of magnesium in the skeleton is about 60% of its total amount in the body [62]. Magnesium is an integral component of apatite crystals, from which it is released during bone resorption [63]. It is also a cofactor of enzymes taking part in numerous metabolic pathways, such as the synthesis of high-energy compounds (ATP), metabolism of lipids, proteins, and nucleic acids, and also calcium metabolism. In the skeleton, magnesium supports the production of hydroxyapatite [64] and mineralization of bone marrow stromal cells [65]. Magnesium also supports the synthesis of vitamin 1,25(OH)2D [62,66]. Magnesium deficiency increases the synthesis of parathormone and osteoclast activity and causes a decrease in osteoblast activity, thereby accelerating the loss of bone mass, which, together with the decline in bone formation, leads to a decrease in the volume of the trabecula and changes in bone microarchitecture, similar to osteomalacea [67]. Even a small decline in the magnesium level below the norm can result in impaired bone growth, skeletal fragility, and osteoporosis [68]. Although mineralization defects have been observed in post-menopausal women and patients with chronic kidney failure following magnesium overload, supplementation with this element is believed to be beneficial to bone health [69]. In the context of these data, the approximately 14% reduction in magnesium content in the case of the diet supplemented with zinc nanoparticles in comparison with the standard diet may pose a significant threat to bone structure, and indicate disturbed magnesium homeostasis, possibly induced not only by the neoplastic process but also by competition (e.g., at the absorption stage) with zinc ions, especially in nano form.

This study showed interesting changes in the level of boron in the femurs. Studies on animals have shown that boron does not accumulate in large quantities in soft tissues, but has a tendency to reach much higher levels in bone tissue [70]. In the bone, it appears exclusively in the mineral part, and manifestations of boron deficiency are nonspecific and include arthritis, loss of bone mass, and osteoporosis [71]. It should be noted that in the present study, only in the case of supplementation with zinc nanoparticles was there a very great increase in the content of this element in the femurs of rats. In the case of the micro form of zinc, its level declined with respect to both the standard diet and the diet with zinc nanoparticles. Many studies have shown a beneficial effect of boron on bone health [70,72]. It stimulates growth of bones and maintains their mechanical properties, as well as the microarchitecture of the trabecular. Appropriate boron levels may be beneficial in arthritis, alleviating pain and discomfort and reducing inflammation [72,73]. It is interesting that boron levels in healthy bones are higher than in people with arthritis [74]. Moreover, arthritis is less common in places where the daily intake of boron in the population is 3–10 mg than in places where it is 1 mg or lower [75]. A low boron level is linked to a decrease in the density of chondrocytes in the proliferative zones of bones. It also exacerbates symptoms of vitamin D and magnesium deficiencies, increasing the amount of calcium excreted in the urine [72,76]. Therefore, supplementation with boron will help to improve bone calcification and decrease calciuria. Boron deficiency can cause changes in the concentrations of other elements involved in bone metabolism, including magnesium, copper, and zinc [67]. Some studies indicate that boron has a beneficial effect on steroid hormones involved in bone metabolism [72]. It has been suggested that boron increases the concentration and effectiveness of 17-oestradiol, improving the density and volume of the trabecular and the density of the bone growth plate [69]. These reports suggest that the significant increase in the boron level observed in our study in the femurs of rats receiving a diet supplemented with zinc nanoparticles in comparison to the standard diet may be favourable to bone tissue metabolism. This may be linked to the facilitation of absorption or possibly to the incorporation of boron in the bone under the influence of zinc nanoparticles. This effect was not observed in the case of zinc microparticles; in fact, there was a substantial decrease in boron content.

Another important change observed in our study in the bones of rats receiving zinc nanoparticles was a substantial reduction in the content of Cd (in contrast with the group receiving zinc microparticles), Tl, V, and U in comparison with the rats receiving a standard diet. Given the highly unfavourable effects of cadmium, this may be a positive change. Cadmium is an extremely toxic element, present in large concentrations in industrial areas. The toxicity of cadmium for bone tissue results from direct and indirect—nephrogenic—mechanisms. Prior to any consequences for the bones, environmental exposure to cadmium affects the kidneys, causing dysfunction of the renal tubules and leading to hypercalciuria and, thus, to a decrease in mineral bone density [69]. All of these factors increase the risk of fractures, osteomalacea, and osteoporosis [77]. Although toxicity affects both sexes, women, especially after menopause, are more susceptible than men [78]. Chronic exposure to cadmium can lead to itai-itai disease, characterized by osteoporosis with osteomalacea and renal tubule dysfunction [79]. Cadmium toxicity is associated with calcium homeostasis, the collagen matrix, and bone cell metabolism [80]. This element can also impair calcium metabolism during osteogenesis, increasing calciuria. The direct mechanism of cadmium toxicity is manifested by its effect on osteoblasts, causing a reduction in their activity and an increase in bone resorption [69]. It can also lead to changes in the collagen matrix, on which bone mineral is deposited, by stimulating osteoclast proliferation and activity. The latest studies indicate that the toxicity of cadmium may also result from its ability to induce oxidative stress [81,82]. Because this element is easily absorbed, it generally accumulates in bones in amounts that are dependent on the natural environment and diet [83]. Its release is most likely beneficial for the functioning of the bone tissue itself, but at the same time it can cause large amounts of cadmium to appear suddenly in the blood serum. Cadmium is known to be a carcinogenic element, and it may perhaps intensify an existing neoplastic process, although it cannot be ruled out that the excess will be efficiently removed from the body.

Our study showed a very large increase in Ag content in the group of rats receiving zinc microparticles in comparison to the standard diet. This is difficult to explain because to date, Ag has not been shown to play an important role in bone tissue. The bactericidal activity of silver has long been known. It mainly involves a reaction with thiol groups of the bacterial cell wall, an increase in the permeability of the cell membrane, disturbances in ion balance, and a destructive effect on DNA [84]. The role of silver in bone tissue function is unknown, but our results indicate the need for research on this subject.

## 5. Summary

In this experiment, we tested whether the presence of breast cancer tumours in a rodent model without metastases (induction of breast tumours by DMBA) and with a modified diet could alter bone mineral composition. The results show that nano and micro zinc supplementation had an effect on the levels of trace elements in the bones of rats with breast cancer. It is also possible that breast tumours may induce signals/release substances into the circulation that cause changes in bone mineral composition before the actual metastasis to the bone tissue, thus preparing the bone tissue for metastasis. The consequences of the changes in the levels of elements are unknown, and this requires further research. A certain limitation of this experiment is the lack of a control group unaffected by DMBA.

## Figures and Tables

**Table 1:** Chemical composition of the diets containing different forms of Zn sources.

Groups	s	m	n
Added Zn	0	4.6 mg/mL	4.6 mg/mL
Total Zn	76.9 mg/kg diet *	230.7 mg/kg diet *	230.7 mg/kg diet *

s—standard group; m—group receiving zinc in microparticles; n—group receiving zinc in nanoparticles. *—average daily standard feed (Labofeed H) intake—12 g.

**Table 2:** Comparison of statistical differences in the body weights and femur weights of rats from different groups.

Groups	s	m	n	*p* Value
Body weight (g)	231.0 ± 13.8	230.1 ± 17.2	230.4 ± 10.2	n.s.
Mass of femur (g)	0.904 ± 0.052 ^a^	0.964 ± 0.05 ^b^	0.951 ± 0.028 ^b^	0.05

Data are shown as mean values ± standard deviation (SD); ^ab^—values with different superscript letters in rows significantly differ at *p* value ≤0.05; n.s.—not significant; s—standard group; m—group receiving zinc in microparticles; n—group receiving zinc in nanoparticles.

**Table 3:** Median, mean, and standard deviation of the elemental content in the bones of rats with cancer receiving various diets (standard, nano-, and microzinc-supplemented).

Groups		s		n		m
Elements (n = 8)	Median	Mean ± SD	Median	Mean ± SD	Median	Mean ± SD
As (ppb)	43.2	45.9 ± 10.02	34.4	38.2 ± 15.04	53.55	53.96 ± 8.892
Ba (ppb)	2999	2980 ± 252	2826	2841 ± 116	2889	2935 ± 24
Cr (ppb)	122.8	123 ± 37	99	102 ± 28	105	108 ± 26
Cu (ppb)	569	535 ± 115	666	747 ± 265	506	532 ± 104
Fe (ppb)	63,791	66,503 ± 12,930	63,896	65,433 ± 9266	69,727	69,766 ± 16,199
K (ppm)	1226	1241 ± 154	1354	1339 ± 79	1391	1348 ± 168
Mn (ppb)	315.8	311 ± 37	322	319 ± 46	294	287 ± 6
Na (ppm)	3947	3874 ± 352	3759	3746 ± 70	3766	3780 ± 141
Ni (ppm)	47.2	37.7 ± 24.0	22.5	27.2 ± 26.2	59.7	47.0 ± 21.5
Rb (ppb)	1483	1491 ± 155	1508	1507 ± 101	1428	1494 ± 254
Se (ppb)	112	107 ± 16	94	95 ± 12	112	108 ± 15
Sr (ppb)	39,026	39,402 ± 2926	39,744	40,169 ± 1839	38,989	38,475 ± 2091

Data are shown as mean values ± standard deviation (SD); s—standard group; m—group receiving zinc in microparticles; n—group receiving zinc in nanoparticles. For the comparison of groups, the Kruskal–Wallis test was performed. There were no statistically significant differences.

## Data Availability

Data is contained within the article.
